# Use of front face fluorescence spectroscopy coupled with multivariate data analysis for monitoring biscuits' quality during aging

**DOI:** 10.1002/fsn3.3032

**Published:** 2022-08-20

**Authors:** Hayet Ghnimi, Romdhane Karoui, Hamadi Attia, Christine Chénè, Monia Ennouri

**Affiliations:** ^1^ University Artois,University Lille, University Littoral Côte d'Opale, University Picardie Jules Verne, University de Liège, INRAE, Junia, UMR‐ T 1158, BioEcoAgro Lens France; ^2^ University Monastir, Higher Institute of Biotechnology of Monastir Monastir Tunisia; ^3^ University Sfax, LR11ES45, National Engineering School of Sfax Sfax Tunisia; ^4^ Adrianor Tilloy Les Mofflaines France; ^5^ University Sfax, LR16IO01, Olive Tree Institute Sfax Tunisia

**Keywords:** biscuits, chemometry, front face fluorescence spectroscopy, oxidation, pomegranate peel extract, storage

## Abstract

In this study, the potentiality of front face fluorescence spectroscopy (FFFS) for the evaluation of the quality of biscuits manufactured with butylated hydroxytoluene and pomegranate peel extract during aging was investigated. By using the principal component analysis, vitamin A and tryptophan spectra allowed a clear discrimination between biscuit samples according to the nature of antioxidants, while fluorescent Maillard reaction products spectra showed clear differentiation between samples according to the storage time. Clear differentiation between biscuits according to the used antioxidants and storage time was achieved by using common components and specific weights analysis. Using partial least‐squares regression, excellent prediction of water activity (*R* ^2^ = 0.95), and L* values (*R* ^2^ = 0.92), and approximate prediction of hardness (*R* ^2^ = 0.78), b* values (*R* ^2^ = 0.74), and moisture content (*R* ^2^ = 0.74) were shown. However, the FFFS failed to predict a* values, primary and secondary oxidation products (*R* ^2^ < 0.6).

## Graphical abstract

Front face fluorescence spectroscopy succeeded in monitoring the quality of biscuits during aging. Common components and specific weights analysis allowed biscuits’ discrimination as a function of used antioxidants and storage time. Partial least‐squares regression succeeded in predicting water activity, moisture content, hardness, and colorimetric data of biscuits.

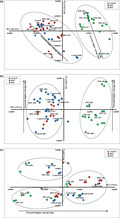

## 1 INTRODUCTION

Cookies or biscuits are very widespread and versatile snack foods occupying a noteworthy position in bakery industry due to their various tastes and attractive properties like longer shelf life, and texture, including vast consumption (Ghnimi et al., 2021). During the manufacturing and the storage of biscuits, many organoleptic, textural, and physicochemical changes may take place. The lipid oxidation, Maillard reaction, and caramelization reactions explicate most of these changes. However, since biscuits contain high amounts of lipids (usually above 20%), lipid oxidation is the most common reaction having negative effects on their quality (Mildner‐Szkudlarz et al., 2009). Oxidative changes are generally connected with higher negative incidence on human health. Therefore, conserving the high quality of biscuits is of wide importance from nutritional and economical points of view, since these snack foods are extensively used and often stored for prolonged periods before eating. For this purpose, the delay in lipid oxidation reaction by adding synthetic antioxidants, such as butylated hydroxytoluene (BHT), butylated hydroxyanisole (BHA), and tert‐butylhydroquinone (TBHQ), has been reported in the literature (Mildner‐Szkudlarz et al., 2009; Nanditha et al., 2009). However, recent investigations have indicated that these synthetic antioxidants might be involved in many health risks (Caleja et al., 2017). Hence, natural antioxidants obtained from fruits and plants have acquired considerable attention, thanks to their efficacy in preventing the oxidation of biscuits (Aly et al., 2021; Caleja et al., 2017; Imeneo et al., 2021; Reddy et al., 2005).

Pomegranate (*Punica granatum* L.) is one of the most important sources of bioactive components that are mainly present in the fruit peels with reported potential health improving properties (Kaderides et al., 2020; Singh et al., 2018). Pomegranate peels are recognized as a good source of phenolic compounds such as phenolic acids, flavonoids, anthocyanins, and so on (El‐Hadary & Ramadan, 2019). That is why, over the past years, many research works have focused on their use in the formulation of biscuits as natural antioxidants (Ismail et al., 2014; Kaderides et al., 2020; Lu et al., 2022; Paul & Bhattacharyya, 2015; Srivastava et al., 2014).

Several techniques, such as physicochemical, textural, colorimetric, gas chromatography, high‐performance liquid chromatography, and sensory analysis, are enhanced over the past decades to assess the quality of biscuits (Ghnimi et al., 2021). Although these analyses have been proved as well‐organized methods, they are expensive, laborious and involve the use of many toxic chemical products. Therefore, fast screening methods are mainly used, nowadays, to monitor lipid oxidation levels in cereals and cereal products. For instance, front face fluorescence spectroscopy (FFFS) is considered as a nondestructive, rapid, and a relatively low‐cost technique for determining the quality parameters of cereal and cereal products (Xue & Tan, 2022). The technique was used for the assessment of the quality of cakes throughout storage by Botosoa et al. (2013) and Nhouchi et al. (2019). In the case of biscuits, researches are limited to the two research studies of Allais et al. (2006) and Edoura‐Gaena et al. (2007) exploring the potential of FFFS to determine the impact of aeration conditions on sensory and physical properties of batters and biscuits. As far as we know, no study has been reported in the literature on the use of FFFS to monitor biscuit aging. So, the aim of this work was to examine, for the first time, the potential of FFFS to monitor the quality of biscuits during storage at 35°C and 65% relative humidity (RH). The impact of the addition of pomegranate peel extract (PPE) compared to BHT to enhance the stability of biscuits during storage was considered. Multidimensional analysis such as principal component analysis (PCA), common components and specific weights analysis (CCSWA), and partial least‐squares regression (PLSR) were applied in order to extract solid information from the data sets.

## 2 MATERIALS AND METHODS

### 2.1 Ingredients

Biscuits were manufactured using wheat flour, crystal sugar, baking powder, and salt purchased from a local supermarket. Palm oil was supplied by a local society (LAND'OR). Pomegranate peel powder (*P. granatum* L.) was bought from a local society (Nopal Tunisie). One hundred grams (100 g) of powdered pomegranate peel was extracted for 2 h with 1 L of distilled water in a laboratory shaker at 60°C. The obtained extract was centrifuged at 2500 *g* at 25°C for 15 min and frozen at −18°C until further use.

### 2.2 Biscuit production

Biscuits were produced following the recipe described previously by Saha et al. (2011). Three batches of biscuits were prepared: control, batch containing BHT, and another one with PPE at 0.02% (lipid weight basis). After baking, all the samples were cooled for 1 h at room temperature, then the biscuits were packed and placed up to 96 days in a dark climate chamber at a temperature of 35°C (Romani et al., 2016) and under a RH of 65%. Biscuits were analyzed in triplicate after 6, 17, 33, 47, 60, and 96 days.

### 2.3 Physicochemical analysis

To determine the moisture content, the samples were ground in a blender (Retsch GM 2000) at 2500 rpm (revolutions per minute) during 40 s at 20°C and ~1 g was weighed in a dish and placed in an oven at 105°C for 24 h. The water activity was measured at 25°C using water activity (aw) meter (Aqualab).

To determine the peroxide value (PV) and thiobarbituric acid reactive substances (TBARS), fat was extracted from biscuits according to the procedure described by the “Association Française de Normalisation” (AFNOR, 1991). Crushed biscuits were combined with a mixture of hexane/isopropanol (3/2; v/v) and stirred at ambient temperature, then the extract was clarified by retaining the puree through a column of Celite and sodium sulfate laid. The obtained filtrate was dried in a rotary evaporator (Buchi, Rotavapor R‐3). The PV of extracted fat was determined according to Nhouchi et al. (2019) and a TBARS assay was performed, as described by Pokorný et al. (1985).

### 2.4 Color determination

The color of the biscuits was measured, for the whole biscuit, with the Minolta Chroma Meter (Model CR‐300, Konica Minolta Sensing Europe) in a CIE Lab system at room temperature (∼20°C). Color values were recorded as L* = lightness (where 0 = black, 100 = white), a* (+a* = redness and −a* = greenness), and b* (+b* = yellowness and −b* = blueness). Total color difference (∆E*) and color saturation (chroma, C*) were determined according to Goswami et al. (2015):

$$∆E*=\sqrt{\;{\left(∆L*\right)}^{2}+{\left(∆a*\right)}^{2}+{\left(∆b*\right)}^{2}}$$

$$C*=\sqrt{\;{\left(a*\right)}^{2}+{\left(b*\right)}^{2}}$$

(∆E*) was calculated with respect to the color at day 06 that is considered as the reference. The (∆E*) values were used to determine whether the total color difference of biscuit samples was appreciable by the human eye (Hassoun & Karoui, 2016):

### 2.5 Texture profile analysis

Texture of biscuit samples was determined using a TA‐XT2 (Stable Micro Systems) texture analyzer equipped with the Exponent Software. Biscuit texture was measured by means of a cutting‐shear test using a blade (type HDP/BSK). The texture conditions were: pretest at 1 mm s^−1^, test speed at 3 mm s^−1^, post test speeds at 10 mm s^−1^, distance at 15 mm, and trigger force at 20 *g*. The maximum force (*g*) was recorded and referred to as the hardness of biscuits.

### 2.6 Fluorescence spectroscopy measurements

Fluorescence spectra were recorded using a FluoroMax‐4 spectrofluorimeter (Jobin Yvon, Horiba). The incidence angle of the excitation radiation was set at 60°. The spectrofluorimeter was equipped with a thermostated cell and the temperature was commanded by a Haake A25, AC 200 temperature controller (Thermo‐Scientific). Ground biscuits were directly placed into a 10 × 10 × 45 mm quartz cell and fluorescence spectra were registered at 20°C. The excitation spectra of vitamin A (252–380 nm) were scanned with the emission wavelength set at 410 nm. The emission spectra of nicotinamide adenine dinucleotide (NADH) (360–600 nm), tryptophan (310–470 nm), and fluorescent Maillard reaction products (FMRP) (380–650 nm) were acquired with the excitation wavelength set at 290, 340, and 360 nm, respectively.

### 2.7 Mathematical analysis data

To determine statistical differences (*p* < .05) between biscuit samples during the storage period, one‐way analysis of variance (ANOVA) with least significant difference (LSD) Fisher's test was used.

To decrease scattering effects and to make a comparison between the samples, a normalization of fluorescence spectra was applied. Indeed, the area under each spectrum was reduced to a value of 1; the peak maximum shift and changes in peak width were considered following this normalization.

First, to investigate differences between the samples, PCA was applied separately to the normalized textural, colorimetric, physicochemical, and fluorescence measurements. The PCA changes the original variables into new axes (principal components [PCs]). Then, CCSWA was applied to the whole data sets, in order to define several data tables obtained for the same sample. With CCSWA, a common space of representation for all the data tables was determined. By projection on the plans defined by each couple of the *CC1*, *CC2…*, *CCn* dimensions, similarity maps of the samples can be drawn (Karoui et al., 2006).

Finally, by applying PLSR the potential of FFFS to predict some chemical parameters was evaluated. So, a statistical model between the treated spectra and biscuit parameters was created. In this study, the PLSR was established for linking the variations in response variables (water activity, moisture content, L*, a*, b*, PV, and TBARS values) to the variations of several predictors (fluorescence intensities). The 54 spectral measurements were divided into calibration (2/3 number of the data set) and validation sets (1/3 number of the data set). While, 36 spectra were used for the establishment of the PLSR statistical models, 18 spectra were used for the established models’ validation. As the successful establishment of the PLSR model consisted of modeling and validation procedures, the leave‐one‐out cross‐validation way was used according to the PLSR modeling procedure. The performance of the PLSR model was evaluated by determining the square of the correlation coefficients of calibration (*R* ^2^_c_) and validation (*R* ^2^_p_), the root mean square error of calibration (RMSEC), the root mean square error of prediction (RMSEP), and the ratio of prediction to deviation (RPD). A good PLSR model exists if the value of the *R* ^2^ is above 0.81, while the RMSEC and RMSEP are as low as possible and the minimum values for RPD are higher than 2 (Karoui et al., 2007).

MATLAB software (Matlab, Version 6.5, Release 12, The MathWorks) was used for performing the PCA and CCSWA, while the Unscramble X (V.10.4, Camo Software AS) software was used to realize PLSR.

## 3 RESULTS AND DISCUSSIONS

### 3.1 Evolution of the physicochemical parameters of biscuits acquired during storage

The evolution of moisture content and water activity of biscuit samples throughout storage are reported in Table 1a. All the fresh biscuits aged 6 days exhibited water activity values in the 0.45–0.51 range, regardless of the recipe suggesting the microbiological stability of these products. The water activity followed a similar trend during storage attaining values of 0.68–0.70 at the 96th day of storage.

**TABLE 1:** Evolution of: (a) water activity and moisture content; (b) peroxide value (PV) and thiobarbituric acid reactive substances (TBARS) values of biscuit samples during storage; and (c) color parameters (L*, a*, b*, C*, and ∆E*)

Storage time (days)	Water activity			Moisture content (%)		
	Control	BHT	PPE	Control	BHT	PPE
*(a)*						
6	0.45^fC^ ± 0.00	0.51^eA^ ± 0.00	0.48^fB^ ± 0.00	6.83^dC^ ± 0.20	7.48^cA^ ± 0.45	7.12^dB^ ± 0.10
17	0.56^eC^ ± 0.00	0.60^dA^ ± 0.00	0.57^eB^ ± 0.00	7.85^cdA^ ± 0.10	8.10^cA^ ± 0.44	8.34^cA^ ± 0.27
33	0.62^dB^ ± 0.00	0.65^cA^ ± 0.00	0.62^dB^ ± 0.00	8.98^bcA^ ± 0.66	9.17^bA^ ± 0.17	8.93^bA^ ± 0.18
47	0.64^cAB^ ± 0.00	0.66^cA^ ± 0.01	0.64^cB^ ± 0.00	8.87^abAB^ ± 0.18	10.25^aA^ ± 0.17	9.34^aB^ ± 0.15
60	0.65^bB^ ± 0.00	0.68^bA^ ± 0.00	0.65^bC^ ± 0.00	9.84^abA^ ± 0.84	9.81^abA^ ± 0.10	8.55^bcA^ ± 0.06
96	0.68^aB^ ± 0.00	0.70^aA^ ± 0.00	0.68^aB^ ± 0.00	9.98^aA^ ± 0.13	9.55^bA^ ± 0.24	9.64^aA^ ± 0.18

Storage time (days)	PV (meq O_2_ kg^−1^ of oil)			TBARS values (mg MDA kg^−1^ of oil)		
	Control	BHT	PPE	Control	BHT	PPE
*(b)*						
6	1.70^eA^ ± 0.13	1.70^bA^ ± 0.013	1.30^bB^ ± 0.13	0.06^abA^ ± 0.00	0.03^bcdB^ ± 0.00	0.03^bB^ ± 0.00
17	2.10^dA^ ± 0.07	1.70^bB^ ± 0.07	1.30^bC^ ± 0.07	0.04^abcA^ ± 0.01	0.03^dB^ ± 0.00	0.03^bB^ ± 0.02
33	2.10^dA^ ± 0.07	1.70^bB^ ± 0.07	1.40^bC^ ± 0.13	0.04^bcA^ ± 0.00	0.05^abB^ ± 0.01	0.03^bC^ ± 0.00
47	3.59^cA^ ± 0.13	2.09^aB^ ± 0.00	1.89^aB^ ± 0.07	0.06^aA^ ± 0.01	0.05^abcA^ ± 0.00	0.05^aA^ ± 0.01
60	4.79^aA^ ± 0.13	2.17^aB^ ± 0.07	2.09^aB^ ± 0.07	0.06^abA^ ± 0.01	0.06^aA^ ± 0.01	0.06^aA^ ± 0.00
96	4.00^bA^ ± 0.00	2.19^aB^ ± 00.0	2.00^aB^ ± 0.13	0.03^cA^ ± 0.00	0.03^cdA^ ± 0.00	0.023^bB^ ± 0.023

Storage time (days)	L*			a*			b*		
	Control	BHT	PPE	Control	BHT	PPE	Control	BHT	PPE
*(c)*									
6	72.32^bB^ ± 0.06	73.43^aA^ ± 0.19	69.82^aC^ ± 0.07	4.63^cB^ ± 0.20	5.28^bA^ ± 0.07	5.41^eA^ ± 0.02	32.16^bcA^ ± 0.05	31.30^fB^ ± 0.27	29.61^fC^ ± 0.02
17	72.35^bB^ ± 0.23	73.10^bA^ ± 0.00	69.22^cC^ ± 0.11	4.17^eC^ ± 0.11	5.31^bB^ ± 0.01	6.16^aA^ ± 0.01	32.02^cA^ ± 0.34	32.19^dA^ ± 0.05	31.06^eB^ ± 0.00
33	72.19^bcB^ ± 0.07	72.32^dA^ ± 0.02	67.57^fC^ ± 0.01	4.55^dC^ ± 0.01	4.92^cB^ ± 0.11	5.92^cA^ ± 0.00	32.30^bcA^ ± 0.24	31.89^eB^ ± 0.04	31.16^dC^ ± 0.04
47	72.05^cB^ ± 0.02	72.30^dA^ ± 0.06	68.55^eC^ ± 0.01	4.86^bC^ ± 0.18	5.51^aB^ ± 0.02	5.76^dA^ ± 0.00	32.52^abB^ ± 0.08	33.44^aA^ ± 0.02	31.63^cC^ ± 0.01
60	72.15^bcB^ ± 0.02	72.78^cA^ ± 0.04	69.00^dC^ ± 0.02	4.94^aC^ ± 0.02	5.05^cB^ ± 0.01	6.11^bA^ ± 0.00	32.72^aA^ ± 0.04	32.59^cB^ ± 0.06	32.28^aC^ ± 0.02
96	72.94^aB^ ± 0.01	73.13^bA^ ± 0.01	69.39^bC^ ± 0.01	4.87^bC^ ± 0.01	5.04^cB^ ± 0.00	5.75^dA^ ± 0.00	32.04^cB^ ± 0.02	33.06^bA^ ± 0.01	31.78^dC^ ± 0.01

Storage time (days)	∆𝐸*			C*		
	Control	BHT	PPE	Control	BHT	PPE
6	–	–	–	32.49^bcA^ ± 0.05	31.75^fB^ ± 0.27	30.10^fC^ ± 0.03
17	0.61^aB^ ± 0.16	0.95^cB^ ± 0.034	1.75^dA^ ± 0.08	32.29^cA^ ± 0.34	32.63^dA^ ± 0.05	31.66^eB^ ± 0.00
33	0.30^bC^ ± 0.07	1.33^bcB^ ± 0.28	2.78^aA^ ± 0.08	32.62^bcA^ ± 0.24	32.27^eA^ ± 0.03	31.72^dB^ ± 0.04
47	0.51^abB^ ± 0.11	2.43^aA^ ± 0.33	2.42^bA^ ± 0.06	32.88^abB^ ± 0.08	33.89^aA^ ± 0.01	32.15^cC^ ± 0.01
60	0.67^aC^ ± 0.08	1.47^bc^ ± 0.31	2.89^aA^ ± 0.03	33.09^aA^ ± 0.04	32.98^cA^ ± 0.07	32.86^aB^ ± 0.02
96	0.68^aC^ ± 0.05	1.80^abB^ ± 0.30	2.25^cA^ ± 0.04	32.41^cB^ ± 0.02	33.47^bA^ ± 0.01	32.30^bC^ ± 0.01

*Note*: Mean values and standard deviations from three replicates are presented. Different small letters (a, b, c, d, e, f) represent statistical differences between different storage days (*p* < .05). Different capital letters (A, B, C) represent statistical differences between different batches for the same storage days (*p* < .05). Control: biscuits produced without antioxidants. BHT: biscuits supplemented with butylated hydroxytoluene at 0.02%. PPE: biscuits supplemented with pomegranate peel extract at 0.02%.

Abbreviations: PV, peroxide value; TBARS, thiobarbituric acid reactive substances.

Regarding moisture content, all biscuits aged 6 days ranged from 6.83% to 7.48% and significantly increased (*p* < .05) with time of storage, reaching levels of ~10%. Our findings are in line with the results of Paciulli et al. (2018), affirming that biscuit absorbed water from the surrounding environment, showing thereby an increase in the moisture content during storage. This increase could be due to the hygroscopic nature of biscuits, the storage time, the RH, the temperature, and the nature of packaging (Romani et al., 2016).

The PV and TBARS values are presented in Table 1b. Regarding the control biscuit, the PV increased from 1.70 to 4.79 meq O_2_ kg^−1^ of oil between the 6th and 60th day of storage. After 60 days of storage, PV levels tended to decrease reaching 4 meq (milliequivalent) O_2_ kg^−1^ of oil after 96 days. This trend could be related to the degradation of primary oxidation products with time of storage. For the biscuits produced with BHT and PPE, the PV of those aged 6 days were 1.70 and 1.30 meq O_2_ kg^−1^ of oil, respectively, and achieved values of 2.19 and 2.00 meq O_2_ kg^−1^ of oil after 96 days, respectively. Calligaris et al. (2008) reported that a bakery product could be considered stable when the PVs do not go over the limit of 10 meq O_2_ kg^−1^ of oil. Therefore, all biscuits kept during 96 days could be considered as highly stable in regard to lipid oxidation.

The TBARS values varied slightly during storage attaining their maxima after 60 days of storage with values of 0.06 mg MA kg^−1^ of oil for the three biscuit formulations. The TBARS values less than 0.576 mg MA kg^−1^ of oil sample indicate that food products are considered fresh (Izzreen & Noriham, 2011). In the present work, all biscuits exhibited TBARS values <0.576 mg MA kg^−1^ of oil, indicating that biscuit samples are tolerable for consumption in agreement with the results obtained with PV (<10 meq O_2_ kg^−1^ of oil).

### 3.2 Evolution of color and texture parameters acquired on biscuits during storage

The changes in L*, b*, and a*values are presented in Table 1c. Significant effects (*p* < .05) of both BHT and PPE on biscuit color were obtained. In fact, samples prepared with BHT were characterized by higher lightness while those supplemented with PPE were characterized by lower lightness. Color analysis presented a rise in red color component (a*) when PPE was added to biscuits (from 4.51 to 5.7). In fact, the main colorants in PPE are anthocyanins characterized by red color (Kaderides et al., 2020). Therefore, the rise in a* value in biscuits supplemented with PPE could be attributed to the presence of anthocyanins in the latter.

Total color difference (∆E*) and chroma (C*) are presented in Table 1b. For the control biscuits, ∆𝐸 * was lower than 1, which indicates that color differences are not obvious to the human eye. For biscuits prepared with BHT and PPE, ∆E* were >1 and <3, except for BHT stored for 17 days (∆𝐸* = 0.95), indicating that color differences are not appreciated by the human eye. Over storage time, while *∆*𝐸* in control biscuits remained stable (*p* > .05), it increased significantly from 0.95 to 2.43 (after 47 days of storage) and from 1.75 to 2.89 (after 60 days of storage) for biscuits formulated with BHT and PPE, respectively.

Regarding Chroma (C*), the lowest values were found in all biscuit samples aged 6 days and significantly increased (*p* < .05) during aging from 32.49 to 33.09 for control samples (60 days), from 31.75 to 33.89 for biscuits supplemented with BHT (47 days), and from 30.10 to 32.86 for biscuits enriched with PPE (60 days), revealing a significantly higher color saturation.

The changes in hardness values of biscuit samples are shown in Figure S1. All biscuit samples showed significant decrease (*p* < .05) in hardness (3575.58 g vs. 2585.25, 3603.09 vs. 2633.18, and 3635.01 vs. 2622.11 g for control, those supplemented with BHT and PPE, respectively, during 96 days of storage). The decrease in hardness could be related to the increase of moisture content. In this regard, a high correlation was found between hardness and moisture content (0.72 < *R* ^2^ < 0.92) (Figure S2a–c), which accords well with the findings of Romani et al. (2016) who reported that the level of moisture increases during storage induced a loss in the biscuit hardness. Similarly, the increase in water activity of the biscuit samples resulted in a decrease of hardness (0.91 < *R* ^2^ < 0.95). Additionally, no difference in hardness (*p* > .05) was found between control biscuits and those produced with antioxidants, in accordance with the observations of Nanditha et al. (2009) who showed that adding BHA to biscuits did not affect the textural quality.

### 3.3 Evolution of fluorescence spectra acquired on biscuits during storage

**FIGURE 1:**
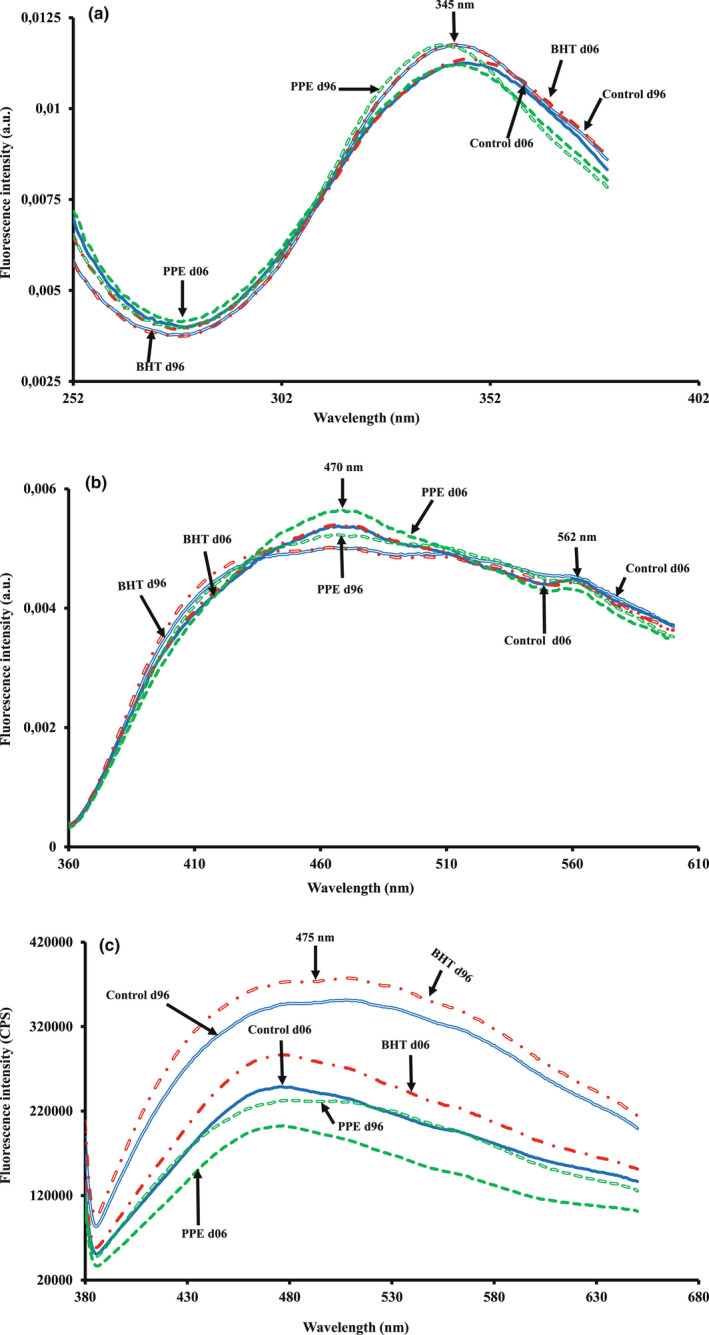
Normalized fluorescence spectra of: vitamin A acquired after emission at 410 nm (a); nicotinamide adenine dinucleotide (NADH) acquired after excitation at 340 nm (b) and raw fluorescence emission spectra of fluorescent Maillard reaction products (FMRP) acquired after excitation at 360 nm recorded (c) on control biscuit, biscuit enriched with butylated hydroxytoluene (BHT) and pomegranate peel extract (PPE) acquired after 6 and 96 days.

The evolution of normalized vitamin A excitation spectra of biscuit samples is presented in Figure 1a. All normalized excitation spectra presented a maximum peak located at ~345 nm. Biscuit samples aged 96 days exhibited the highest fluorescence intensity at ~345 nm, while those aged 6 days had the lowest one. The maximum fluorescence intensity varied according to the aging period. These variations could be due to the difference in the composition of fatty acid, and thus, the triglycerides’ physical state in the fat globule (Karoui et al., 2005; Najib et al., 2020; Nhouchi et al., 2019).

The NADH emission spectra of biscuits presented two maxima at ∼470 and ∼562 nm (Figure 1b). Fresh samples supplemented with PPE aged 6 days exhibited the highest fluorescence intensity at ∼470 nm, followed by control batch and those supplemented with BHT. For all biscuit samples, a significant decrease in the fluorescence intensity was observed at ∼470 nm with the increase of storage time, while an opposite trend was noted at ∼562 nm. The decrease in the fluorescence intensity at ∼470 nm could be related to the chemical reaction occurring during storage. Our observations are in accordance with Allais et al. (2006) who showed that NADH spectra are very sensitive to the batter aging and technological process. In this context, Dufour et al. (2003) have reported that the oxidation of the cytoplasm cell, by increasing storage time, induced the transformation of NADH into NAD^+^ which modifies the shape of the fluorescence spectra. The overall increase in the intensity of fluorescence at ~562 nm throughout storage could result from various fluorescent compounds. Precisely, lipid oxidation products can interact with peptides, amino acids, proteins, nucleic acids, phospholipids, and deoxyribonucleic acid (DNA), producing fluorescent products during storage (Hassoun & Karoui, 2016).

The normalized spectra of tryptophan exhibited a peak at ∼392 nm (data not shown) in accordance with the observations of Nhouchi et al. (2019) who have demonstrated that tryptophan acquired on pound cake exhibits a maximum at ∼394 nm.

An example of FMRP emission spectra, presented in Figure 1c., showed that the shape of emission spectra exhibited maxima located at ∼475 nm. During storage, an increase in the fluorescence intensity was observed. This could be due to the formation of advanced glycation end‐products (AGEs). Indeed, Amadori compounds developed in the initial stage of the Maillard reaction are not fluorescent, but in advanced Maillard reaction stages, these compounds by forming cross‐links with adjacent proteins or with other amino groups lead to the development of fluorescent polymeric aggregates. The fluorescence intensity of these compounds is used as a marker of the yield of Maillard reaction (Bosch et al., 2007; Matiacevich et al., 2005). For instance, Matiacevich et al. (2005) have reported that the range of emission wavelengths of AGEs has extended up to 470 nm. Our findings are in accordance with those of Bosch et al. (2007) who pointed out a gradual increase in free and total AGEs in infant formula during storage.

### 3.4 Discrimination of biscuit samples based on their fluorescence spectra recorded during storage

**FIGURE 2:**
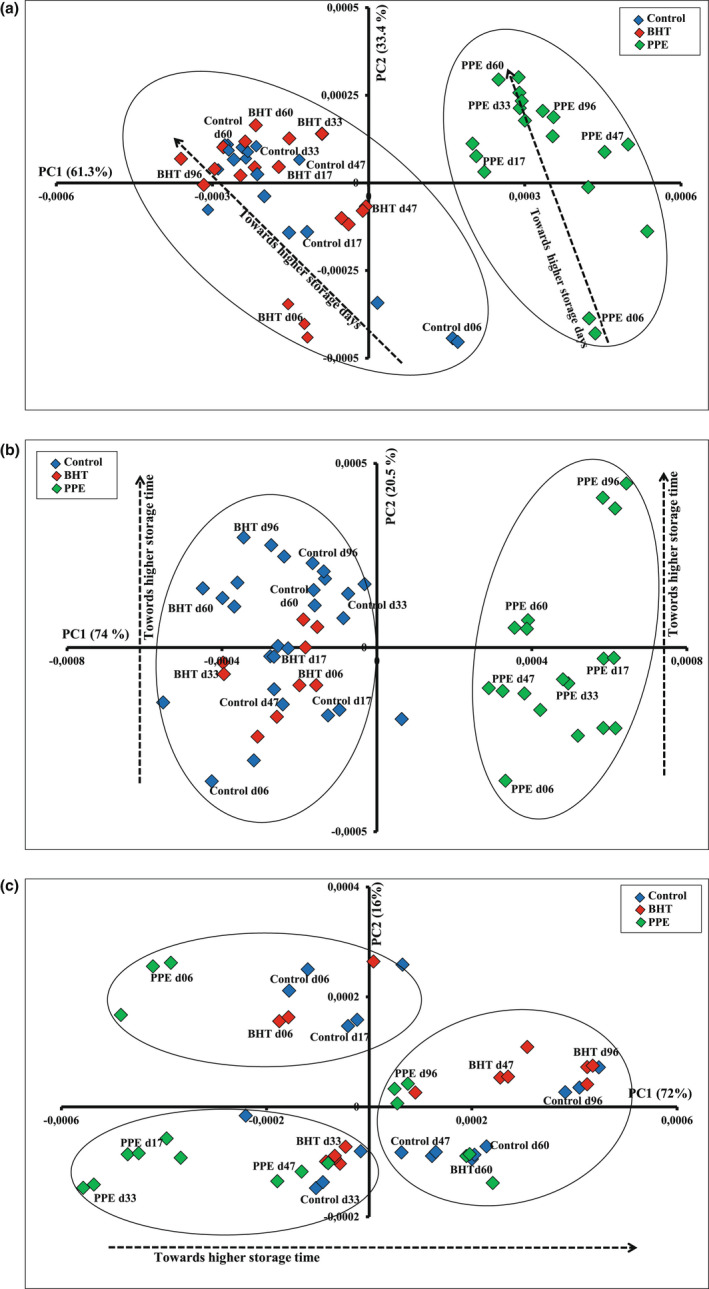
Principal component analysis (PCA) similarity map defined by the principal components 1 and 2 of vitamin A (a), tryptophan (b), and fluorescent Maillard reaction products (FMRP) (c) recorded on control biscuits, and biscuits enriched with butylated hydroxytoluene (BHT) and pomegranate peel extract (PPE) during storage.

The similarity map defined by first principal component (PC1) and second principal component (PC2) (61.3% and 33.4% of the total variance, respectively) of the PCA applied to the vitamin A spectra (Figure 2a) presented a clear discrimination of biscuit samples according to their aging time and the used antioxidants. Indeed, the PC1 mainly allowed a differentiation between samples according to the antioxidant type. Biscuits prepared with PPE were situated on the positive side of the PC1, whereas biscuits prepared with BHT were grouped with the control samples and presented negative scores on PC1. Meanwhile, PC2 discriminated biscuit samples according to their aging time. In fact, control biscuits aged 6 and 17 days had negative scores according to PC2, while those aged 33 days and more were positioned on the positive side. Besides, biscuits with two antioxidants type aged 17 days or more (except those formulated with BHT aged 47 days) exhibited mostly positive scores with PC2, while those aged 6 days showed negative score values. All biscuit samples exhibited similar trends, indicating that similar phenomena occurred during storage. We can conclude that vitamin A spectrum could determine the freshness of biscuit. This is in line with the results of Botosoa et al. (2013) who found that vitamin A is a good indicator for the assessment of sponge cake during storage.

Concerning the PCA applied to NADH, the similarity map defined by PC1 and PC2 (62.3% and 27% of the total variance, respectively) did not show a clear discrimination of samples according to their aging time and the used antioxidants (data not shown). Regarding the PCA performed on tryptophan spectra (Figure 2b), the map defined by PCs1 and 2 (74% and 20.5% of the total variance, respectively) divided the samples into two groups. The first group (group 1) represented the biscuits enriched with PPE, while the second one (group 2) consisted mostly of the control biscuits and those supplemented with BHT. A clear differentiation was shown between these two groups since group 1 is located on the positive side of PC1, while group 2 is positioned on the negative side. With respect to PC2, a clear discrimination of biscuit samples as a function of their storage time was observed. Biscuit samples stored for 60 and 96 days had positive values, while almost all the other samples (age < 60 days) exhibited negative values. Therefore, it can be concluded that PC1 discriminated samples according to their recipes, while PC2 allowed the differentiation of samples according to their storage period.

The map defined by PC1 and PC2 (72% and 16% of the total variance, respectively) of the PCA applied to FMRP showed a clear discrimination between biscuits as a function to their aging time (Figure 2c). Indeed, the PC1 divided samples into three groups: the first one consisting of samples aged 6 days, the second one belonging to samples aged 17 and 33 days, and the last one containing samples aged 60 and 96 days. Groups 1 and 2 exhibited negative values, while the third group had positive values.

### 3.5 Joint analysis of fluorescence spectra by applying common components and specific weights analysis

**FIGURE 3:**
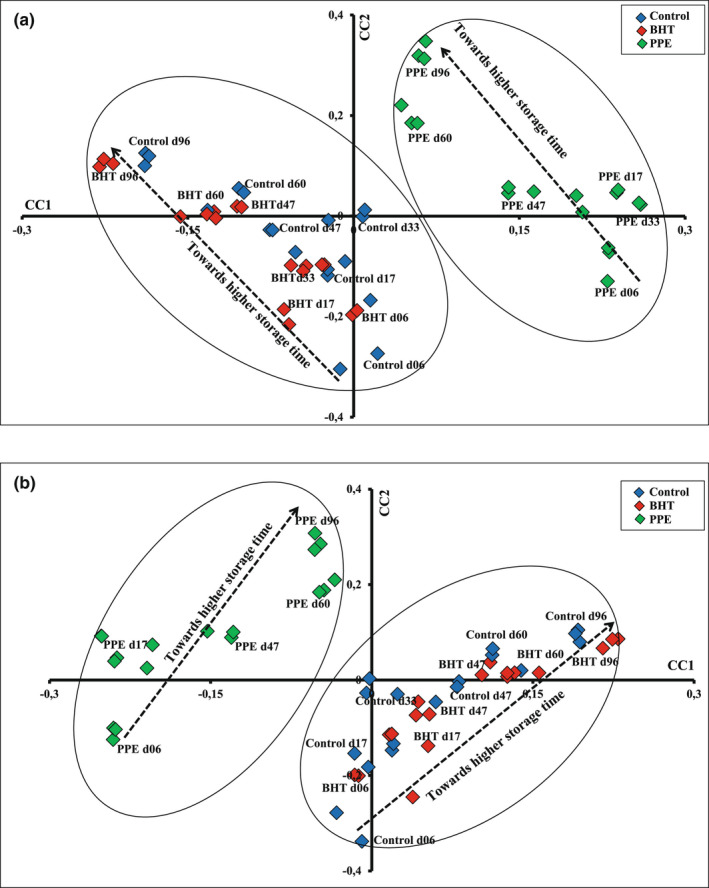
Common component and specific weights analysis (CCWSA) determined by the CC1 and CC2 applied to the: (a) four normalized data sets containing vitamin A, nicotinamide adenine dinucleotide (NADH), tryptophan, and fluorescent Maillard reaction products (FMRP) fluorescence spectra; and (b) vitamin A, NADH, tryptophan, and FMRP fluorescence spectra, physicochemical, textural, and colorimetric measurements recorded on control biscuit, and biscuits enriched with butylated hydroxytoluen (BHT) and pomegranate peel extract (PPE) during storage.

The CCSWA technique was applied to normalized vitamin A, NADH, tryptophan, and FMRP spectra. The similarity map defined by the first two common components *CC1* and *CC2* is shown in Figure 3a. A clear discrimination between biscuit samples regarding the nature of the used antioxidants was observed. Indeed, the *CC1* divided the biscuit samples into two groups. Biscuits with PPE are localized on the positive site of CC1, while control biscuits and those enriched with BHT showed negative score values according to CC1. The CC2 showed a clear differentiation between biscuit samples as a function of time storage: (i) for biscuits supplemented with PPE, samples aged 6 days had negative score values while those aged more than 6 days had positive values; and (ii) control biscuits and biscuits enriched with BHT aged 6–47 days had negative score values, whereas those stored for 60 and 96 days showed mostly positive score values.

To get a better awareness of the correlation between the data at macroscopic and molecular levels, CCSWA was applied to the fluorescence, physicochemical, textural, and colorimetric measurements (Figure 3b). Indeed, biscuits enriched with PPE had negative score values according to CC1, while control and biscuits supplemented with BHT presented positive values. Regarding CC2, samples were differentiated according to their storage time, coordinated with the CCSWA applied to the fluorescence data.

The similarity maps derived from the two CCSWA (Figure 3a,b) were quite similar and divided biscuits into two groups, suggesting a high correlation between the textural, physicochemical, colorimetric, and fluorescence data sets. The phenomenon noted at the macroscopic and the molecular levels is associated with the changes occurring throughout storage and with the nature of antioxidant. In addition, it was reported that the changes occurring at the molecular level made a reflection at the macroscopic one (Karoui, 2018).

### 3.6 Prediction of biscuits’ properties using PLSR

A summary of PLSR model performances on raw and preprocessed fluorescence data is shown in Table 2. The PLSR models were used to predict water activity, moisture content, colorimetric parameters (L*, a*, and b*), and chemical parameters (PV and TBARS).

**TABLE 2:** Cross‐validation results of: (a) water activity and moisture content; (b) color parameters (L*, a*, b*); and (c) peroxide value (PV) and thiobarbituric acid reactive substances (TBARS) values of biscuit using partial least‐squares regression (PLSR)

Biscuit properties	Data	Data pretreatment	Calibration set			Prediction set		
			$R_{C}^{2}$	RMSEC	RPD	$R_{P}^{2}$	RMSEP	RPD
*(a)*								
Water activity	Vitamin A	Raw data	0.94	0.017	4.08	0.889	0.043	3
		Normalization	0.967	0.013	5.5	0.952	0.02	4.56
	NADH	Raw data	0.899	0.022	3.15	0.69	0.043	1.8
		Normalization	0.984	0.009	7.91	0.896	0.025	3.1
	Tryptophan	Raw data	0.916	0.021	3.45	0.781	0.038	2.14
		Normalization	0.98	0.01	7.07	0.939	0.021	4.05
	FMRP	Raw data	0.883	0.024	2.92	0.664	0.049	1.73
		Normalization	0.95	0.016	4.47	0.812	0.033	2.31
Moisture content	Vitamin A	Raw data	0.815	0.459	2.32	0.605	0.459	1.59
		Normalization	0.775	0.506	2.11	0.695	0.728	1.81
	NADH	Raw data	0.712	0.572	1.86	0.655	0.645	1.70
		Normalization	0.74	0.544	1.96	0.737	0.675	1.95
	Tryptophan	Raw data	0.583	0.688	1.55	0.565	0.687	1.52
		Normalization	0.652	0.629	1.7	0.652	0.64	1.7
	FMRP	Raw data	0.614	0.663	1.61	0.569	0.726	1.52
		Normalization	0.689	0.594	1.79	0.631	0.646	1.65
Hardness	Vitamin A	Raw data	0.715	204.87	1.87	0.681	264.1	1.77
		Normalization	0.643	229.47	1.67	0.735	245.34	1.94
	NADH	Raw data	0.969	67.63	5.68	0.727	288.91	1.91
		Normalization	0.965	71.99	5.35	0.686	292.99	1.78
	Tryptophan	Raw data	0.562	253.97	1.51	0.783	229.67	2.15
		Normalization	0.953	82.83	4.61	0.512	322.77	1.43
	FMRP	Raw data	0.748	192.68	1.99	0.676	276.71	1.76
		Normalization	0.754	190.42	2.02	0.675	309.21	1.75

*(b)*								
L*	Vitamin A	Raw data	0.896	0.459	3.1	0.918	0.459	3.49
		Normalization	0.923	0.502	3.6	0.877	0.636	2.85
	NADH	Raw data	0.987	0.21	8.77	0.837	0.789	2.48
		Normalization	0.988	0.2	9.13	0.87	0.688	2.77
	Tryptophan	Raw data	0.884	0.618	2.94	0.652	0.64	1.7
		Normalization	0.927	0.491	3.7	0.905	0.588	3.24
	FMRP	Raw data	0.682	1.022	1.77	0.629	1.13	1.64
		Normalization	0.74	0.924	1.96	0.699	1.003	1.82
a*	Vitamin A	Raw data	0.902	0.168	3.19	0.605	0.365	1.59
		Normalization	0.568	0.353	1.52	0.496	0.395	1.41
	NADH	Raw data	0.938	0.134	4.02	0.539	0.399	1.47
		Normalization	0.958	0.116	4.88	0.556	0.391	1.5
	Tryptophan	Raw data	0.498	0.38	1.41	0.478	0.411	1.38
		Normalization	0.485	0.385	1.39	0.535	0.38	1.47
	FMRP	Raw data	0.829	0.222	2.42	0.493	0.439	1.4
		Normalization	0.821	0.227	2.36	0.359	0.561	1.25
b*	Vitamin A	Raw data	0.721	0.45	1.89	0.517	0.78	1.44
		Normalization	0.836	0.345	2.47	0.538	0.659	1.47
	NADH	Raw data	0.88	0.295	2.89	0.516	0.609	1.44
		Normalization	0.809	0.372	2.29	0.74	0.445	1.96
	Tryptophan	Raw data	0.895	0.275	3.09	0.605	0.536	1.59
		Normalization	0.9	0.269	3.16	0.686	0.471	1.78
	FMRP	Raw data	0.807	0.373	2.28	0.504	0.653	1.42
		Normalization	0.873	0.303	2.81	0.686	0.51	1.78

*(c)*								
PV	Vitamin A	Raw data	0.411	0.729	1.3	0.284	0.766	1.18
		Normalization	0.324	0.781	1.22	0.3	0.745	1.2
	NADH	Raw data	0.976	0.146	6.45	0.386	0.729	1.28
		Normalization	0.403	0.734	1.29	0.326	0.313	1.22
	Tryptophan	Raw data	0.769	0.456	2.08	0.199	0.898	1.12
		Normalization	0.691	0.528	1.8	0.172	0.92	1.1
	FMRP	Raw data	0.658	0.555	1.71	0.335	0.79	1.23
		Normalization	0.443	0.709	1.34	0.313	0.755	1.21
TBARS value	Vitamin A	Raw data	0.361	0.011	1.25	0.191	0.016	1.11
		Normalization	0.029	0.014	1.01	0.056	0.017	1.03
	NADH	Raw data	0.944	0.003	4.23	0.342	0.014	1.23
		Normalization	0.895	0.004	3.09	0.269	0.015	1.17
	Tryptophan	Raw data	0.318	0.012	1.21	0.09	0.017	1.05
		Normalization	0.898	0.004	3.13	0.113	0.018	1.06
	FMRP	Raw data	0.829	0.006	2.42	0.157	0.008	1.09
		Normalization	0.9	0.004	3.16	0.084	0.017	1.04

Abbreviations: d RPD, ratio of prediction to deviation; FMRP, Fluorescent Maillard reaction products; NADH, nicotinamide adenine dinucleotide; PV, peroxide value; RMSEC, root mean square error of calibration; RMSEP, root mean square error of prediction; TBARS, thiobarbituric acid reactive substances.

Based on the calibration data sets, a prediction with high accuracy of water activity was observed since $R_{C}^{2}$ ranging between 0.883 and 0.984, RPD ranging between 2.92 and 7.91, and RMSEC were very small (<0.03). Good results for moisture content ($R_{c}^{2}$ = 0.815, RMSEC = 0.506, RPD = 2.32) and hardness ($R_{c}^{2}$ = 0.89, RPD = 2.85) were also obtained. Excellent predictions of L* ($R_{c}^{2}$ =0.988, RMSEC = 0.02, RPD = 9.13) and a* ($R_{c}^{2}$ = 0.958, RMSEC = 0.116, RPD = 4.88), and good prediction of b* ($R_{c}^{2}$ = 0.9, RMSEC = 0.269, RPD = 3.16) were observed. Regarding chemical parameters, excellent results were obtained for PV ($R_{c}^{2}$ = 0.976, RMSEC = 0.146, RPD = 6.45) and TBARS values ($R_{c}^{2}$ = 0.944, RMSEC = 0.003, RPD = 4.23).

**FIGURE 4:**
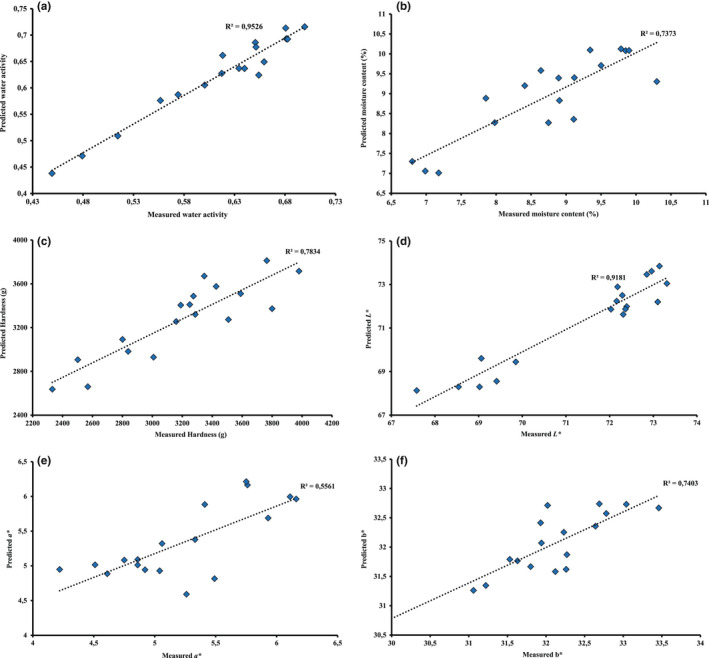
Scatter plots of measured versus predicted of: water activity (a), moisture content (b), hardness (c), L* value (d), a* value (e), and b* value (f) obtained for biscuit samples with full cross‐validation after partial least‐squares regression (PLSR).

Although our findings reveal good results obtained with calibration set, they are not sufficient for eventual conclusion. Thus, cross‐validation method was used to perceive the model validation and the result is relatively successful (Table 2). According to the prediction data, the best result of prediction of water activity was obtained by applying PLSR on the raw vitamin A and normalized tryptophan data since $R_{P}^{2}$ values were 0.952 and 0.939, RMSEP were 0.02 and 0.021, and RPD were 4.56 and 4.05, respectively (Table 2a; Figure 4a). These findings are in agreement with those of Ozbekova and Kulmyrzaev (2019) who pointed out that applying PLSR on tryptophan data predicted water activity in rice with $R_{P}^{2}$ = 0.74 and RMSEP = 0.512, and $R_{P}^{2}$ = 0.82 and RMSEP = 0.427, respectively. In the same way, the obtained results for the moisture content prediction models presented a satisfactory result with normalized NADH spectra ($R_{P}^{2}$ = 0.737; RMSEP = 0.675; RPD = 1.95) (Table 2a; Figure 4b).

Concerning hardness, the best prediction was obtained by applying PLSR on raw tryptophan data since $R_{p}^{2}$ of 0.783 and RPD of 2.15 were observed (Table 2a; Figure 4c) which was consistent with the previous results of Allais et al. (2006) who found a correlation between tryptophan spectra and hardness of biscuit batters with *R* ^2^ >0.98.

In the case of colorimetric parameters, the best prediction of L* value was obtained with raw vitamin A spectrum ($R_{p}^{2}$ = 0.918; RMSEP = 0.459; RPD = 3.49) (Table 2b; Figure 4d), while the best prediction of a* value was achieved with raw NADH spectra ($R_{p}^{2}$ = 0.837; RMSEP = 0.789; RPD = 2.48) (Table 2b; Figure 4e), and an approximate prediction of b* was observed with normalized NADH spectra ($R_{p}^{2}$ = 0.740; RMSEP = 0.445; RPD = 1.96) (Table 2b; Figure 4f).

Regarding PV and TBARS, unsuccessful results were obtained since very low *R* ^2^ of prediction was obtained ($R_{p}^{2}$ < .4) (Table 2c). These findings are in line with the results of Nhouchi et al. (2019) who showed a poorer prediction of PV and TBARS value with fluorescence spectroscopy.

As a result, this study revealed the potential of FFFS to predict water activity, moisture content, hardness, and colorimetric parameters with an excellent precision. However, an unsuccessful prediction of chemical parameters was observed.

## 4 CONCLUSIONS

To best knowledge of the authors, this work is the first one assessing the quality of biscuits during storage using FFFS coupled with chemometrics. The storage time induced an increase in the fluorescence intensity of vitamin A and FMRP, and a decrease in NADH fluorescence intensity. Using PCA, the vitamin A and tryptophan spectra allowed a clear discrimination between biscuit samples according to the used antioxidants, while the FMRP spectra showed a good differentiation according to the storage time. The most interesting findings were found by applying CCSWA to the fluorescence spectra data sets, since a clear differentiation between biscuit samples according to the presence of antioxidants and storage time was achieved. Applying CCSWA to the whole data sets showed that the information pertaining to the molecular and macroscopic levels is complementary. Using PLSR, FFFS is appropriate to determine the water activity, moisture content, hardness, and colorimetric data of biscuits, while poorer prediction was obtained for primary and secondary oxidation indicators. Thus, FFFS coupled with multivariate data analysis could be used and proposed as a fast and nondestructive method for the assessment of the quality of biscuit throughout storage. However, this work is only a preliminary study and further investigations using other fats (like rapeseed oil) in the formulation of biscuit are needed to offer robust models.

## Supporting information


Figure S1
Figure S2

## Data Availability

The data that support the findings of this study are available from the corresponding author upon reasonable request.
