# Supplementary material for: Use of front face fluorescence spectroscopy coupled with multivariate data analysis for monitoring biscuits' quality during aging
Source: Food Sci Nutr. 2022 Aug 20;10(12):4380–93. doi: 10.1002/fsn3.3032 (PMC9731564; doi:10.1002/fsn3.3032)
Supplement: Supplementary file 1 — Figure S1 Figure S2 [file FSN3-10-4380-s001.docx]

**Supplementary figure captions:**

Fig. S1. Evolution of the texture parameters of biscuit samples during storage

Fig. S2. Correlation between hardness and water activity (♦) and moisture content (♢) of control biscuit (a), biscuit made with BHT (b) and biscuit made with PPE (c).

**Fig. S1.**

*Different small letters (a, b, c, d, e) represent statistical differences between different storage days (p < 0.05).*

*Different capital letters (A, B, C) represent statistical differences between different batches for the same storage days (p < 0.05).*

*Control: biscuits produced without antioxidants*

*BHT: biscuits supplemented with BHT at 0.02%*

*PPE: biscuits supplemented with pomegranate peel extract* *at 0.02%*

**Fig. S2.a**

**Fig. S2.b**

**Fig. S2.c**
